# Continuity in intuition and insight: from real to naturalistic virtual environment

**DOI:** 10.1038/s41598-021-81532-w

**Published:** 2021-01-21

**Authors:** M. Eskinazi, I. Giannopulu

**Affiliations:** 1 Université de Paris, Paris, France; 2 Interdisciplinary Centre for the Artificial Mind (iCAM), FSD, Bond University, 14 University Drive, Gold Coast, QLD 4226 Australia

**Keywords:** Human behaviour, Neuroscience, Psychology

## Abstract

Intuition and insight can be deployed on the same continuum. Intuition is the unconscious ability to create links between information; insight is a process by which a sudden comprehension and resolution of a situation arises (i.e. euréka). In the present study, real and virtual environments were used to trigger intuition and insight. The study hypothesised that immersion in real primed environments would facilitate the emergence of intuition and insight in a virtual environment. Forty nine healthy participants were randomly assigned to two groups: “primed” and “non primed.” “Primed” participants were immersed in a real environment with olfactory and visual cues; “non primed” participants did not receive any cues. All participants were exposed to a 3D naturalistic virtual environment which represented a district in Paris via a Head Mounted Display (HMD). Locations presented in the virtual scene (i.e. café places) were related to both olfactory and visual primes (i.e. café) and were based on the continuity between real and virtual environments. Once immersed in the virtual environment, all participants were instructed to use their intuition to envision the selected locations during which Skin Conductance Responses (SCRs) and verbal declarations were recorded. When initiation (a) and immersion (b) phases in the virtual environment were considered, “primed” participants had higher SCRs during the immersion phase than the initiation phase in the virtual environment. They showed higher SRCs during the first part of the virtual immersion than “non primed” participants. During the phenomenological interview, “primed” participants reported a higher number of correct intuitive answers than “non primed” participants. Moreover, “primed” participants “with” insight had higher SCRs during real environment immersion than “primed” participants “without” insight. The findings are consistent with the idea that intuitive decisions in various tasks are based on the activation of pre-existing knowledge, which is unconsciously retrieved, but nevertheless can elicit an intuitive impression of coherence and can generate insight.

## Introduction

In everyday real life-situations, we are constantly and rapidly settling issues and making decisions based on incomplete information. Unconscious feelings and thoughts, which facilitate these decisions are essentially qualified as intuition. The most common paradigm to study intuition (or intuitive answers) is to investigate the semantic coherence of word-triads^1–4^. The typical empirical finding is that individuals are usually accurate in achieving this task. This suggests an undisputed continuity in the intrinsic perceptual processes of information based on semantic process. A neglected question is the analysis of the strategies people use to create links between cues which will trigger insight, in real vs artificial environments. Based on bi-modal visual and olfactory signals, the objective of the present study was to analyse intuition and insight on a continuum starting from a real environment and extending to a naturalistic virtual environment.

Intuition is defined as “a sense of knowing without knowing how one knows”^5^. It involves a complete and expeditious feeling of coherence extracted by signals in the environment^6,7^. The most accepted idea is that intuition is inherently associated with pre-established knowledge informed by implicit memory and learning^8,9^. Linked to these implicit processes, intuition performs in an unconscious automatic mode from which emerge feelings and thoughts that can give rise to conscious verbal and/or nonverbal declarations. In other words, intuition can be conceived of as a continuum starting from automatic and implicit processes based on sensory visual, and/or olfactory inputs and leading to organised verbal/nonverbal decisions. This is consistent with Bower’s et al.’s^1^, model that proposes a two-stage process: during the first stage, named the *guiding stage*, one assembles verbal and/or nonverbal cues and progressively combines them into a coherent but not precise scheme; during the second stage, entitled the *integrative stage*, combined information is associated with mnemonic implicit traces and is able to propose an option once a given perceptual threshold is reached. It is evident that everything is organised at an unconscious level where representations of the provided information are organised, thoughts develop and can be gradually transferred to consciousness. The question of whether intuition and implicit memory are the same or distinct processes is an open one. Current theoretical approaches propose that intuition depends on implicit memory, but it is specific per se^10^.

In priming, an individual is first in contact with a verbal or nonverbal information without being aware of the relevance of whether it is treated unconsciously or not. Then, the same individual is in contact with the same information (e.g. a triangle), or a conceptual variation of it (e.g. triangle vs geometry). Both situations involve coherent judgements that necessitate, on one hand, identifying the consistencies between the perceived information and on the other, establishing mental representations based not only on provided information but also on information activated in memory. Therefore, one responds based on feeling without being aware of the cognitive processes and the cues that are used to infer a specific answer. With this in mind, it seems that intuition is linked to knowledge and priming to feelings. Based on unconscious analysis, priming can be considered an implicit knowledge form.

The association between intuition and insight is still unclear^7^. Intuition and insight seem to be qualitatively distinct. Insight is reported to be a discontinuous process resulting from a mental restructuring mechanism. As such, insight is linked to the *representational change theory* according to which previous information and knowledge has a negative effect on the generation of solutions^11^. Both prior information and experience introduce a kind of stalemate that provokes inappropriate resolution which, in turn, constitutes the requisite condition for representational modifications, i.e. the restructuring process, that gives rise to insight^12^. The representational approach eliminates the intuitive precursor of insight. Supposedly similar, intuition and insight can be expressed on the same continuum^13,14^. Intuition signifies solutions based on tacit representations and can presage insight; insight designates a sudden comprehension and resolution of a situation, (i.e. euréka), by combining various elements^14^. As such, the mechanism that paves the way from intuition to insight gradually develops from a tacit information towards an explicit comprehension and expression. Explicit representations progressively emanate from implicit knowledge built on environmental cues. In that context, accumulated implicit knowledge anticipates explicit representations; implicit knowledge is the unconscious messenger of insight. According to this approach, intuition and insight are intrinsically interlaced. However, the cognitive and physiological mechanisms that authorise the enactment from felt intuition to substantiate insight are still unclear. For a better understanding of these mechanisms, intuition and insight need to be explored within the same procedure and participants via both physiological and cognitive processes. This is the overarching research aim of the present study.

Studies have shown that the neural components of intuition implicate a specific part of the prefrontal cortex, the anterior medial orbitofrontal cortex (OFC)^14^, which is considered a rapid detector of nonverbal information^15^ since it intervenes in early stages of object recognition (e.g. drawings of nameable objects). A kind of global and imprecise representation appears to be generated and involved in a top-down processing towards neural areas essentially facilitating actions. Luu et al.^16^ suggested that the OFC intervenes in order to facilitate preliminary perception and recognition processes of a visual stimulus (i.e. judgment of line fragments). When participants are, therefore, invited to perform visual coherence judgments, OFC activation seems to be dependent not only on physical stimulus characteristics, but also on task specifications^17^. Other parts of the prefrontal cortex (PFC) are also activated when participants are involved in insightful problems^18,19^ indicating high order cognitive processes such as semantic recuperation of information. However, the prefrontal cortex is not the only structure implicated in intuitive judgments of consistency and insight. Using triads intuitive tasks inviting the participants to perform semantic judgments, Ilg et al.^20^ reported activations not only in the OFC but in bilateral associative areas of the inferior parietal cortex and also in the right superior temporal cortex. Superior temporal cortex is also involved in insight^18^. In the same vein, when participants were instructed to solve verbal problems and indicate whether they solved them with or without insight, fMRI revealed specific metabolic activity in the anterior superior temporal gyrus of the right hemisphere. Interestingly, EEG recordings revealed a rapid activation of high-gamma oscillations (i.e. superior to 30 Hz) in the same neural area starting 0.3 s prior to insight answer^21^. Furthermore, the literature about priming and implicit memory suggests bilateral occipitotemporal regions indicated blood flow reductions during a word-stem completion task^22^.

Insightful solutions have been described to provoke subtle neural modifications in hippocampi, parahippocampi gyri, anterior and posterior cingulate cortex^23,24^ and amygdala^19^. Such areas are involved in various cognitive processes including memory, motivation, and emotion^25^. In addition, changes comprising the ventral segmental area (VTA), nucleus accumbens (NAcc) and caudate nucleus underlining the affective aspects of insight have been identified^26^.

From the above, it is clear that there are diverging behavioural and neural correlates of insight and intuition, which is probably due to their inconsistent operationalisation. Only a few studies reported automatic correlates (i.e. involuntary neurophysiological correlates) with respect to the autonomic nervous system and suggested that both intuition and insight are associated with modification in electrodermal activity. Specifically, when healthy participants were invited to perform problem-solving tasks, their electrodermal responses were greater for insight trials compared to non-insight ones^27^. Similarly, the unconscious analysis of a familiar face in healthy participants^28,29^ and prosopagnosic patients^30^ involving intuition are associated with an increase in electrodermal activity. Electrodermal activity per se seems to be the only autonomic marker that reflects sympathetic neuronal activity^31^ which is associated with an unconscious process based not only on intuition^32^, but also on memory recall and decision-making processes^33^. Electrodermal activity is dependent on cortical and subcortical area functioning. Increases in electrodermal activity have been found to be associated with direct activation of cingulate, lateral prefrontal cortex, medial and middle temporal gyrus^33,34^ and with the insula, pre and post central gyri, putamen and parietal cortices^35^.

**Figure 1:**
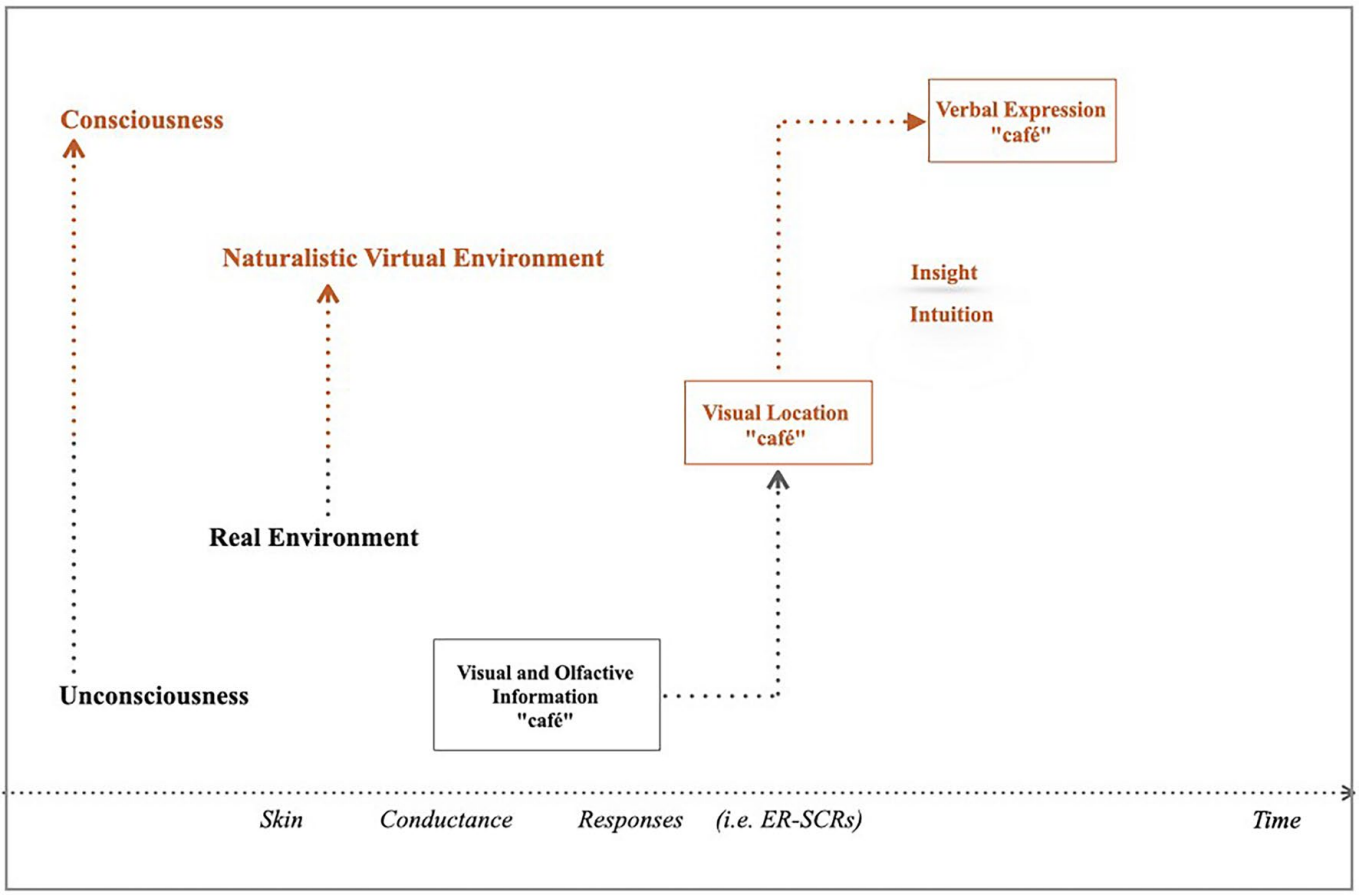
Representation of intuition and insight continuum within real (dark gray colour) and naturalistic virtual (orange colour) environments. Healthy participants were immersed in a real environment which provided visual and olfactive information associated with "café" and "café drink" and the visual location of "café shops" in a naturalistic virtual environment (i.e. a Parisian district). Visual and olfactive information was displayed to engender coherent implicit representations in the real environment, afford continuity and facilitate the emergence of intuition and insight (i.e. euréka) in the virtual environment. Autonomic (unconscious) activity mirrored by related event Skin Conductance Responses were recorded during the whole immersion (both in real and naturalistic virtual environments); verbal (conscious) expressions were considered during the virtual immersion.

In the present study, it was considered that the continuity between sensory information (i.e. visual-olfactory-visual) would facilitate the emergence of intuition and insight. To create a sense of continuity, in terms of “being there”^36–38^, eliciting natural reactions^39,40^, healthy participants were immersed in a virtual naturalistic environment after having been immersed in a real environment. A repeated bi-modal design, both visual and olfactory, associated with café places afforded continuity between real and virtual environments. First, posters of a Parisian café place (i.e. visual prime) together with real coffee cups and odours (i.e. visual and olfactory primes) were presented to trigger coherent unconscious representations in the real environment. Then, participants were immersed in a virtual environment showing a Parisian district with many shops including cafés (i.e. visual prime). All participants were verbally instructed to use their intuition to solve a specific problem: figure out the appropriate places (i.e. café(s)), and name them when they passed by. To that end, an experimental group (with visual and olfactory primes) was compared to a control group (without any visual and/or olfactory primes). The general hypothesis was that based on the bi-modal sensory information (visual and olfactory) which affords continuity, the immersion in the real environment (with primes) would facilitate the emergence of intuition and insight in a virtual environment (Fig. 1).

## Method

**Participants.** Forty nine adult volunteers participated in the study. Twenty three (10 males and 13 females) were designated as “primed” participants; twenty six (12 males and 14 females) were “non primed” participants. Their age was 29 years old on average (std 4.1 years). Both groups were immersed in a virtual environment via a Head Mounted Display (HDM). All participants were equally recruited from two Universities in terms of gender, cultural and socio-economical status. They were all from middle and high socioeconomic background. All participants had normal or corrected-to-normal vision and no history of vestibular, cardiac or neurological disorders of spatial perception and orientation. All individuals gave their informed consent both verbal and in written for their participation in the study and data analysis. However, they did not allow us to release the individual data.The study was approved by the CNRS ethics committee, local ethic committees of both universities, and conformed to the declaration of Helsinki 2.0. Anonymity was guaranteed.

### Visual environment and Experimental devices

A Head Mounted Display (HMD) adapted for two identical Smartphones was used to display the virtual environment. The field of vision (FOV) of the smartphone was 50.9° diagonal at full overplay. The HMD was fitted with two visual lenses with FOV of 100°. The virtual environment was a 3D replica of a real urban scenario pre-recorded on video named “Louvre-Rivoli” scenery^41–44^. Representing a part of the 1st district of Paris between the Louvre museum and the Opera, the 3D scenery is a naturalistic virtual environment. The environment started at the Pyramid square and continued down Pyramid street until the intersection with St Honoré street; then left on St Honoré street in the direction of Palais Royal Square, down Rohan street and right on Rivoli street to arrive at the Pyramid square straight ahead. In that district, there were 29 different locations: 12 restaurants, 6 cafés, 4 hotels, 2 public places, 2 museums, 2 banks and 1 chemist. The environment was controlled by a MacBook Pro computer.

An Affectiva Q Sensor bracelet was used to measure electrodermal activity across the skin. This measurement involved a tiny amount of direct current between two electrodes in contact with the skin^45–47^. The unit of measurement was microSiemens (μS).

### Procedure

The procedure comprised of four phases: (a) immersion in real environment (i.e. ImRE); (b) initiation into the virtual environment (i.e. InVE); (c) immersion in virtual environment (i.e. ImVE); (d) phenomenological interview.

### Immersion in real environment (i.e. ImRE)

**Figure 2:**
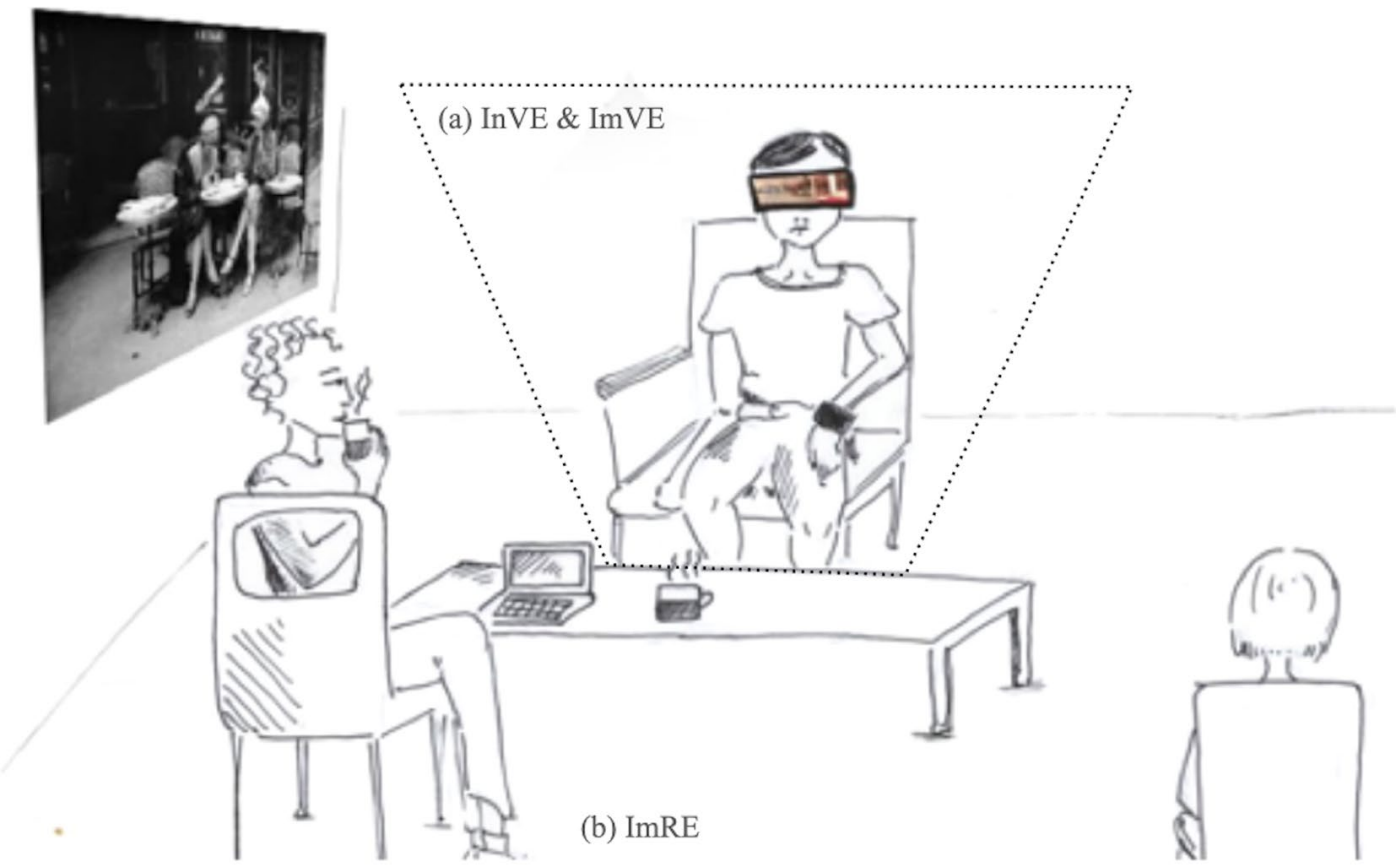
Example of real environment immersion (ImRE) with olfactive and visual information for "primed" participants (designed by Cécile). Note that "primed" participants were equipped with the HMD device when initiated and immersed into the virtual environment (InVE and ImVE respectively) (**a**) they did not wear the HMD device when immersed in the real environment (**b**). The configuration (with and without HMD) was the same for "non primed" participants.

All participants were randomly assigned to the “primed” or “non primed” group. The “primed” participants were immersed in a real environment enriched by visual information and olfactory information. First, “primed” participants were led to the experimental room through a specific corridor where they had the possibility to perceive the posters with “Café scenes” in Paris, hung up on the walls (i.e. visual prime). In order to avoid consciously noticing the posters, the experimenter spoke with the participants while walking along the corridor, after which, both the experimenter and the participant entered the experimental room. The experimental room was composed of visual information associated with a café on a poster, and olfactive information was produced by cups filled with coffee put on a table (i.e. visual and olfactory primes). Two experimenters were present in the room. Twice during the immersion in the real environment (ImRE), one experimenter drank coffee in front of each participant (Fig. 2).

The “non primed” participants were immersed in the same real environment that was neutral (i.e. no visual information in the corridor, no visual and olfactory information, that is posters and coffee odours, no information associated with café places, and no cups in the experimental room). All “non primed” participants were immersed in the same virtual environment as the “primed” participants.

When experimenters and participants were in the experimental room, the electrodermal activity (i.e. baseline recording) was recorded for 1 min in silence for each “primed” and “non primed” participant with the Affectiva Q sensor bracelet.

### Initiation to virtual environment (i.e. InVE)

In the initiation phase, both “primed” and “non primed” participants were immersed in the 3D naturalistic virtual environment “Louvre-Rivoli" via the HMD for 3 min. Each participant was seated on a comfortable chair, with the participant’s back and shoulders in contact with the seat back. Each participant was asked to orient his/her head straight ahead. As soon as the participant’s head was in the correct position and after the participant declared that s/he was ready, the virtual environment was presented on the HMD’s screen. The HMD had built-in stereo headphones through which pink noise was delivered. The presentation of the virtual environment signalled the beginning of the experiment, with the participant sitting correctly and looking at the middle of the virtual environment. The electrodermal activity of each participant was recorded during the immersion phase (3 min). The participants were informed that the stationary environment would move and during the scene’s motion they would be able to visit a Parisian district. Participants were told to observe the environment. The participants were immersed in an optokinetic virtual environment.

### Immersion in virtual environment (i.e. ImVE)

**Figure 3:**
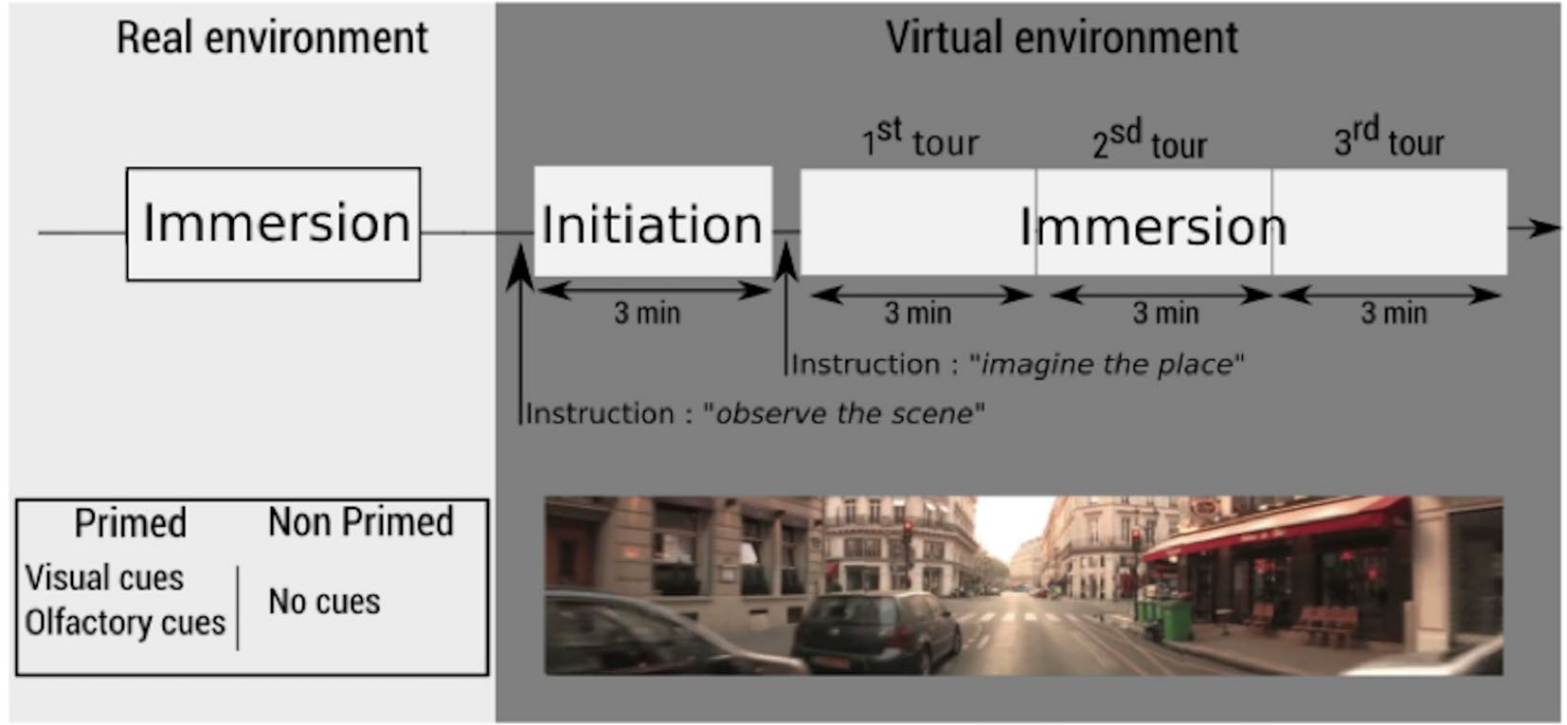
Schematic representation of the experimental procedure and naturalistic virtual environment^41–44^. Participants have been through immersion into real environment (ImRE), initiation into virtual environment (InVE) and immersion into virtual environment (ImVE). “Primed" and “non primed” participants had been immersed into the real environment “with” primes and “without” primes respectively. Each participant performed three consecutive tours when immersed into the virtual environment (ImVE).

During the immersion in the virtual environment, each participant was in the same physical situation as in the “initiation” (InVE) phase and immersed in the same 3D virtual environment. Each participant was given three trials of 3 min each. The inter-trial interval was approximatively 60 s. Each participant was informed again that s/he would be immersed within a virtual environment that represented a part of the 1st district of Paris. It was also explained that in the district some places had been selected along the streets. The participant was instructed to use his/her intuition to figure out the selection of the places, and indicate them verbally (i.e. name them) when s/he passed by. In order to ensure that the participants had understood the task, each participant was asked to repeat the instruction to the experimenter. The electrodermal activity of each participant was recorded during each trial. The total duration of the immersion in the virtual environment was approximatively 12 min (Fig. 3).

### Phenomenological interview

By the end of the experiment, one of the experimenters, always the same, conducted a structured phenomenological interview. Both "primed" and "non primed" participants were asked the same questions, regarding their mental strategies^13^. More particularly, the participants were asked: (a) what they thought once immersed in the virtual environment; (b) what they did to find the solution; (c) how they found the solution; (d) how they understood that the solution was "café" (e) what was/were their strategies to figure out the place. The aim of this interview was to comprehend how participants answered during the virtual immersion (ImVE), on what criteria they based their decisions about which places they had selected, and if they had an “insight” of the solution or not.

### Data analysis

#### Pre-processing

Data analysis was carried out in Matlab 2019a. Prior to the analysis, skin conductance data was converted back to a waveform signal with 125 Hz time resolution. First a low-pass filter with a cut-off frequency of 5 Hz was applied in order to eliminate high-frequency noise. Afterwards a high-pass filter (frequency = 0.05 Hz) performed the separation of the tonic and phasic part of the signal. The cut-off frequency was chosen according to the typical characteristics of the electrodermal activity^48^. For additional noise reduction in the phasic part, the signal was once more low-pass filtered with a cut-off frequency of 5 Hz. This method allowed us to select for analysis only the rapid phasic components, isolating Event Related Skin Conductance Responses (ER-SCRs)^49–51^. The time series was then *z*-transformed and all values were transformed into absolute values to account for between-subjects variance in ER-SCR amplitude, which can be due to peripheral factors such as skin properties. ER-SCRs will referred to as SCR in the following sections.

### Statistical analysis

The Skin Conductance Responses (SCRs) of each participant (“primed” and “non primed”) were recorded in three phases: “immersion in real environment (i.e. ImRE)”, “initiation in virtual environment (i.e. InVE)” and “immersion in virtual environment (i.e. ImVE)”. In the “ImVE” phase, three tours were performed and SCRs were recorded for each tour. Data were collected during each phase (ImRE, InVE and ImVE), and each tour during the “ImVE” phase at a sampling rate of 480 data points per minute resulting in 5760 data points per participant. The total number of data points was 132 480 for the “primed” group, and 149 760 for the “non primed” group. Data assumption controls were conducted using IBM SPSS Statistics 25 software to determine the appropriateness of analysis. Linearity was assessed via visual inspection of a scatterplot matrix and evidence of a linear relationship was not met, thus indicating a violation of this assumption. Sphericity was assessed via Mauchly’s test with a Greenhouse–Geisser epsilon (ε) and a significant value of 0.301 indicating a violation of the assumption. Normality was assessed via Shapiro–Wilk test. All data distributions gave a significance of level less than 0.05 indicating a violation of the assumption of normality. Note that violations of assumptions influence Type I and Type II errors resulting in erroneous inferential measures^63^. Based on the above, the Friedman’s two-way test of variance by ranks, which is a non-parametric alternative to the repeated measures ANOVA, was chosen to compare SCR medians^52^ between each phase for both “primed” and “non primed” participants. Mann–Whitney test was carried out to compare SCR medians between "primed" and "non primed” participants. In addition, Yate’s chi-square test was used to compare the frequencies of “primed” and “non primed” participants “with insight”.

## Results

### Physiological results

Under the hypothesis that immersion in the real environment with primes (i.e. ImRE) would facilitate the emergence of intuition and insight in the virtual environment (i.e. ImVE), it was expected that Skin Conductance Responses (SCRs) would be higher for “primed” than “non primed” participants. Note that all participants reported self-motion once immersed in the virtual environment. None of them experienced motion sickness or similar symptoms.

**Figure 4:**
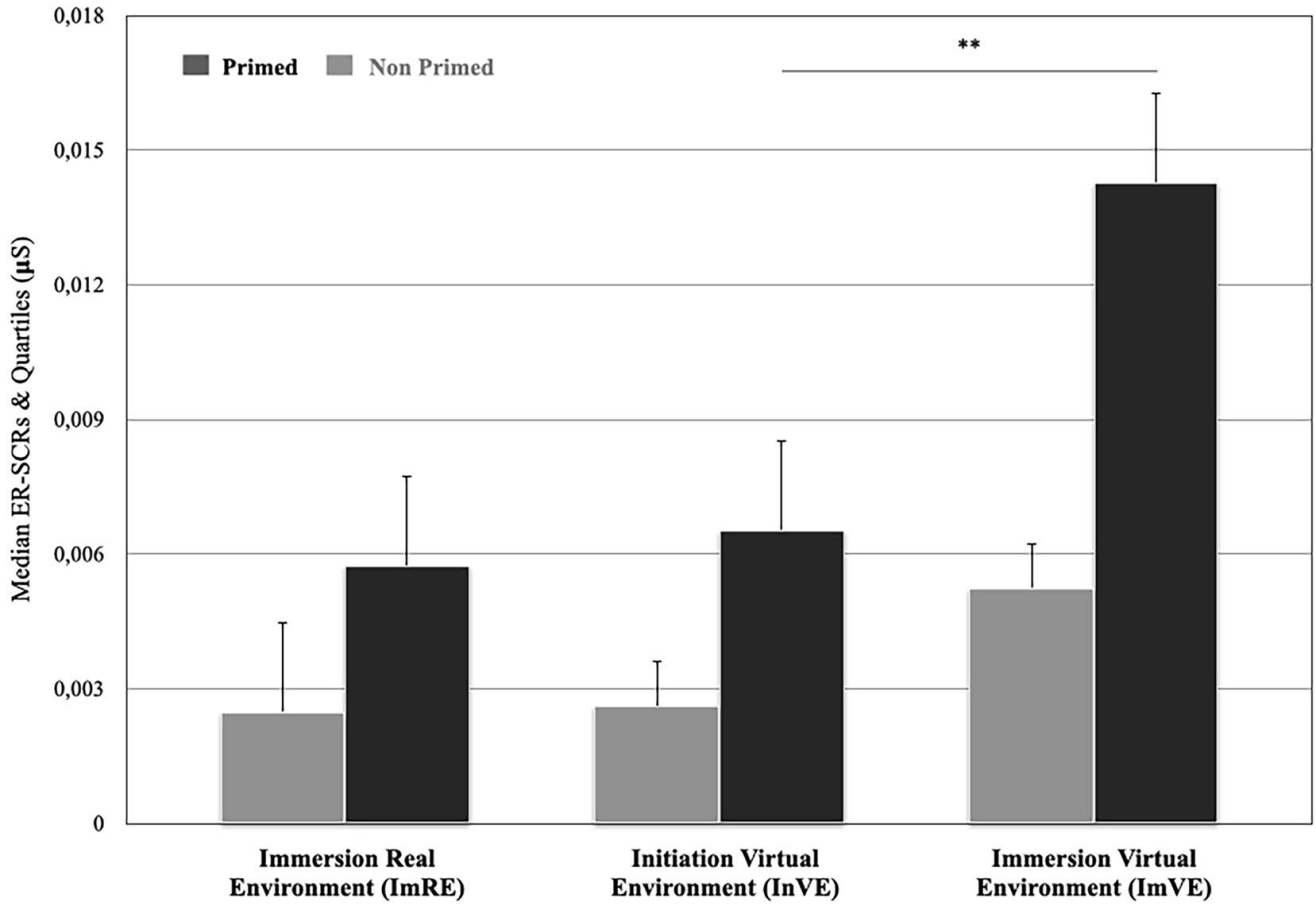
Median SCR comparisons between immersion into real environment (ImRE); initiation into virtual environment (InVE); and immersion into virtual environment (ImVE) for “primed" and “non primed” participants (**p < 0.001).

At the inter-individual level, results showed that the median SCRs did not significantly differ between “primed” and “non primed” participants when immersed in real (i.e. ImRE) and in virtual environments (i.e. ImVE) (Mann–Whitney U = 279, *p* = 0.688, for ImRE; and U = 256, *p* = 0.389 for ImVE) (Fig. 4). More particularly, the median SCRs for “primed” and “non primed” participants were *Mdn* = 0.005 and *Mdn* = 0*.*002 for the ImRE and *Mdn* = 0.002 and *Mdn* = 0.014, for the ImVE respectively. Additionally, the median SCRs were similar when “primed” and “non primed” participants were initiated in the virtual environment (i.e. InVE) (Mann–Whitney U = 278, *p* = 0.674) (Mdn = 0.0026, and Mdn = 0.0065 respectively).

At intra-individual level with “primed” participants, the median SCRs were higher in the ImVE phase (Mdn = 0.014) than in InVE phase (Mdn = 0.002) (χ^2^ (2) = 8.696, *p* = 0.013) and similar between ImRE and InVE phase (χ^2^ (2) = 1.087, *p* = 0.297 with Mdn = 0.005 and Mdn = 0.014 for each phase respectively) and between ImRE (Mdn = 0.005), and ImVE phase (Mdn = 0.014) (χ^2^ (2) = 1.087, *p* = 0.297). Regarding “non primed” participants, the median SCRs did not significantly differ between ImRE (Mdn = 0.002) and InVE phase (Mdn = 0.006), ImRE (Mdn = 0.002) and ImVE phase (Mdn = 0.005) and InVE (Mdn = 0.006) and ImVE phase (Mdn = 0.005) (χ^2^ (2) = 4.846, *p* = 0.089).

With regard to physiological state, only the Immersion in Virtual Environment phase (ImVE) was analysed. According to the hypothesis, if there is a continuity between intuition and insight, the repetition of tours would modify the SRCs of the “primed” participants. Both intra-individual and inter-individual levels were analysed.

**Figure 5:**
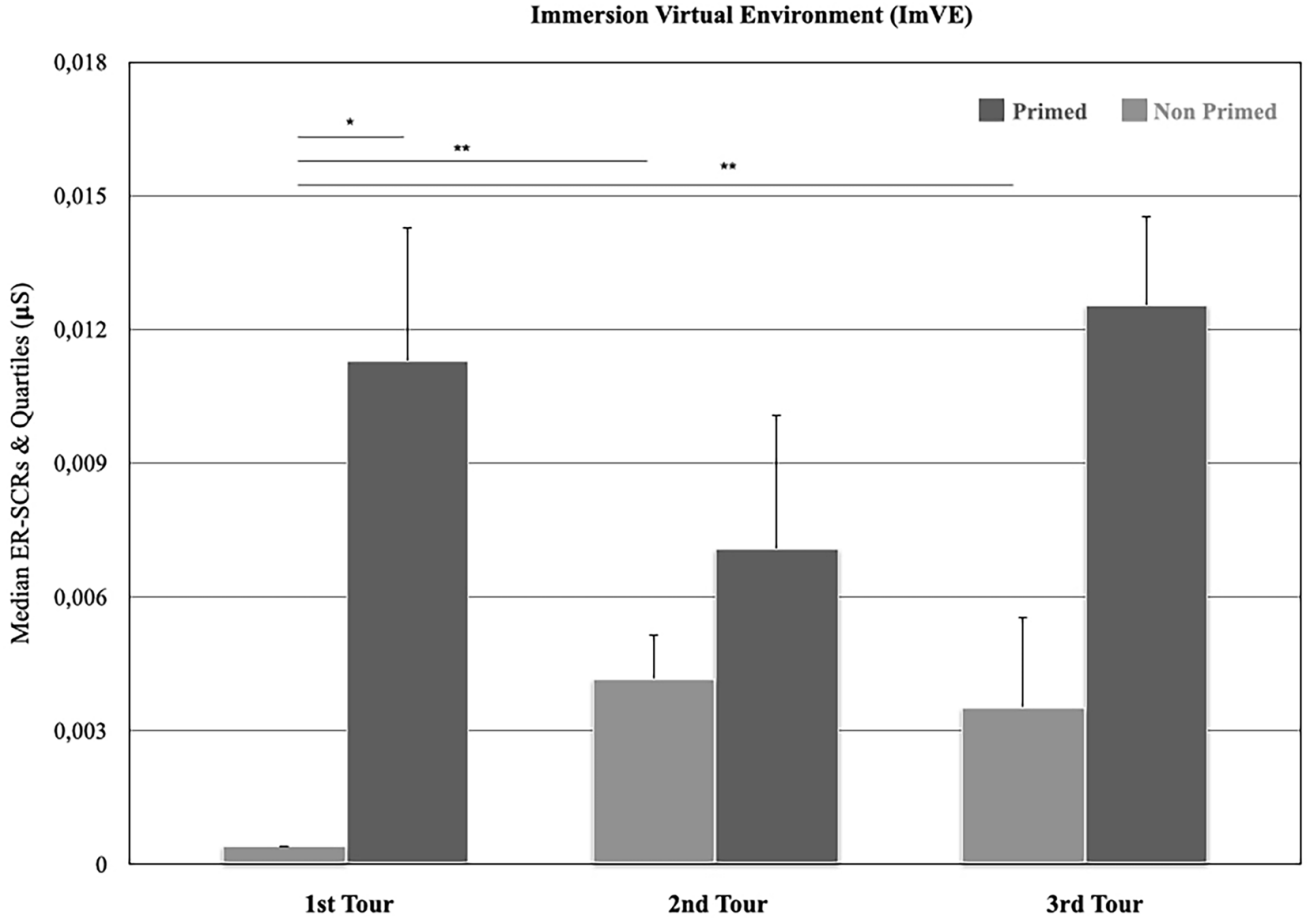
Median SCR comparisons between “primed” and “non primed” participants during the immersion into virtual environment (ImVE) for the 1st, 2nd and 3rd tours (*p < 0.05 , **p < 0.001).

First, the median SCRs within each tour inter-individually were compared (Fig. 5). The statistical analysis showed that during the first tour, the median SCRs were higher for the “primed” (*Mdn* = 0.011) than the “non primed” participants (*Mdn* = 0.000) (Mann–Whitney U = 144.5, *p* = 0.002) but did not significantly differ between “primed” and “non primed" participants for the second (*Mdn* = 0.007 vs *Mdn* = 0.004) and the third tour (*Mdn* = 0.012 vs *Mdn* = 0.003) (Mann–Whitney U = 322, *p* = 0.645 and Mann–Whitney U = 233, *p* = 0.186 respectively). After, the median SCRs between the three tours intra-individually were compared. For the “primed” participants, the median SCRs were similar between the first (Mdn = 0.01), the second (Mdn = 0.007)*,* and the third tour (Mdn = 0.01)*,* (χ^2^ (2) = 3.217, *p* = 0.200). For “non primed” participants, the median SCRs were lower in the first (Mdn = 0.0004)*,* than in the second tour (Mdn = 0.004)*,* (χ^2^ (2) = 15,385, *p* = 0.000), and the third tour (Mdn = 0.003) (χ^2^ (2) = 12.462 p = 0.000) but similar between the second (Mdn = 0.004)*,* and the third tour (Mdn = 0.003)*,* (χ^2^ (2) = 0.000, *p* > 0.99).

**Figure 6:**
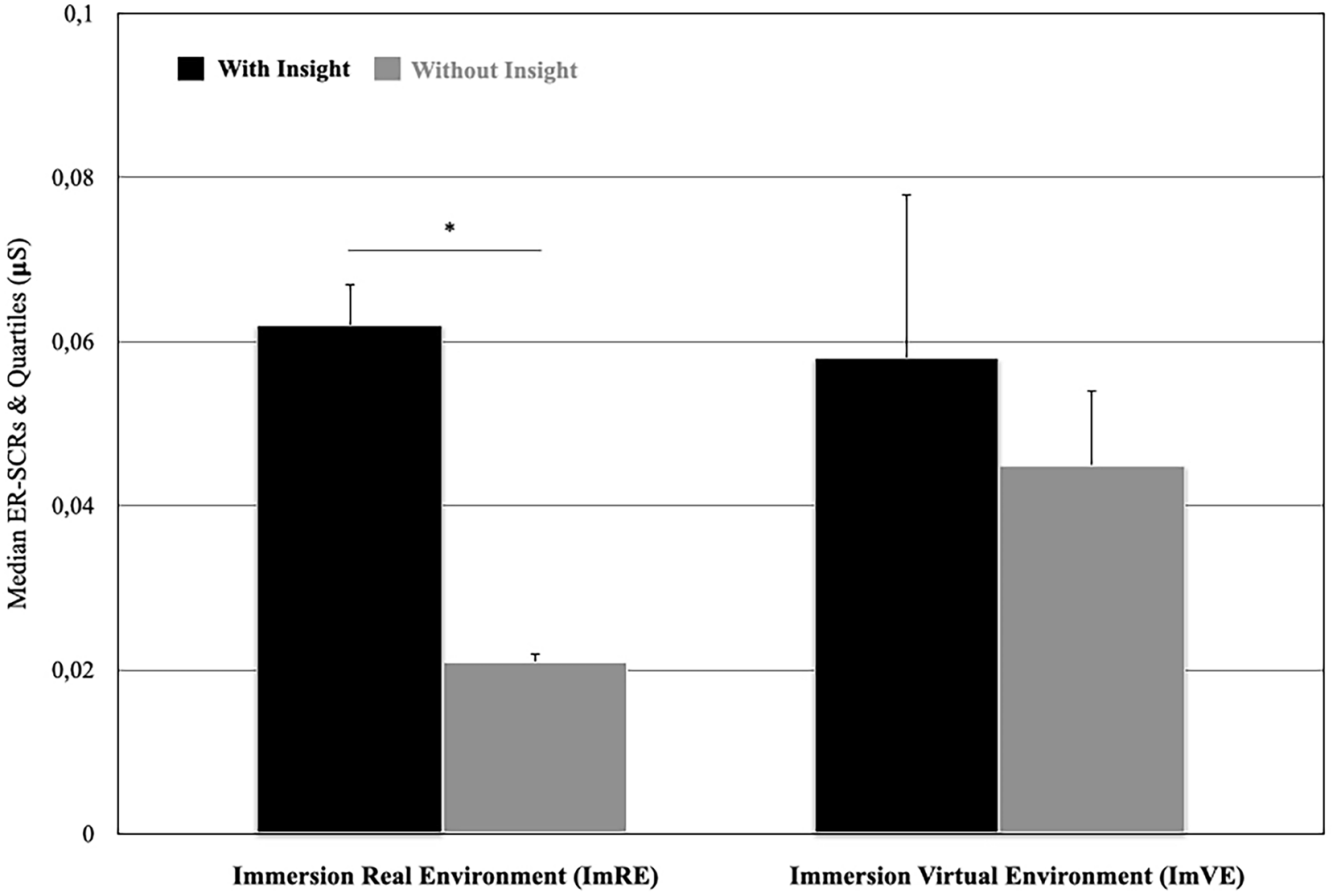
Median SCR comparisons for "primed" participants “with” and “without” insight during immersion into real (ImRE) and virtual (ImVE) environments (*p < 0.05).

Regarding “insight”, it was hypothesised that “primed” participants “with insight” of the intuitive question would have higher SCRs, than “primed” participants “without insight” (both in ImRE and ImVR phases) (Fig. 6). Only the SCRs of participants who verbally declared that “*they knew what the place was*” from the beginning of the experiment (ImRE) were analysed. The Wilcoxon test showed that the median SCRs of the primed participants “with insight” was higher (Mdn = 0.0624) than the median SCR of the participants “without insight” (Mdn = 0.021) (T = 97, *p* = 0.0316) in the ImRE phase but they were similar in the ImVE phase (Mdn = 0.058) for the “primed” participants “with” insight (Mdn = 0.045) and for those “without" insight (Wilcoxon T = 154, *p* = 0.58).

### Behavioural results

**Figure 7:**
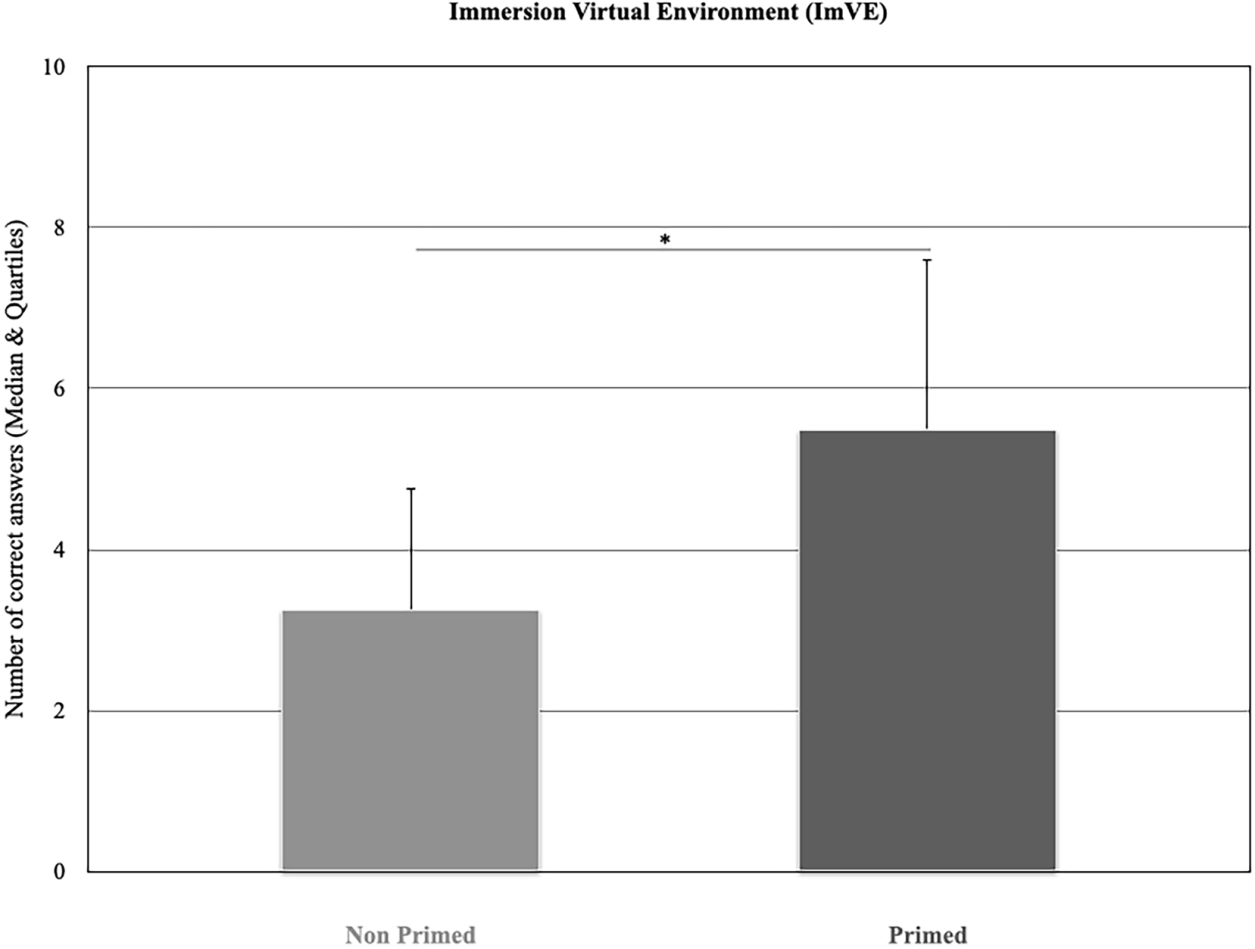
Median number of responses associated with “café” location provided by “primed” and “non primed” participants when immersed into the virtual environment (ImVE) (*p < 0.05).

According to the criteria, only decisions directly associated with the "cafés" places were considered. More precisely, only “cafés” or “café-bar” were the correct solutions. All other solutions, e.g. shops, banks, restaurants, museums and hotels were disregarded. Note that in the virtual environment, there were 29 different locations: 12 restaurants, 6 coffees shops, 4 hotels, 2 public places, 2 museums, 2 banks and 1 chemist. With regard to the hypothesis, if there was continuity between intuition and insight, the correct reported intuitive responses would be in favour of “primed” participants. Consistently, the results showed that the median number of correct decisions was higher for “primed” (Mdn = 5.5) than “non primed” (Mdn = 3.25) participants (Mann–Whitney U = 126, *p* = 0.032) **(**Fig. 7).

A phenomenological interview was used to access participant experience to understand how “primed” and “non primed” participants made their decisions. To that end, participants were invited to explain the strategies they used to verbally produce their decisions during the virtual immersion. All the participants declared that they tried to “reasonably” infer the place (i.e. café). They explained that they based their answers on place location and visual information including colours. Only three out of twenty six “non primed” participants had an “insight” about the situation (i.e. thought that “café” was the place to name), but twenty out of twenty three “primed” participants were “persuaded” and “knew” that the solution was “café” but they could not explain how they “knew” it. Yate’s chi-squared test was calculated comparing the frequency of “primed” and “non primed” participants “with insight”. The results revealed that “primed” participants were more likely to get experience insight (71%) than “non primed” participants (11%) (Yate’s chi-square test = 76.8, *p* < 0.0001).

## Discussion

As far as is known, the current study is the first study that analyses intuition and insight within the same paradigm and the same population through a continuum starting from a real and ending in a naturalistic virtual environment. To that end, healthy participants were immersed in a real primed environment generated by bi-modal information, i.e. visual and olfactory, and then presented unimodal visual information in a naturalistic virtual environment which required them to identify and name a specific location. Bi-modal visual and olfactory information was included in the real environment and was conceptually linked to the café places presented in the virtual environments. Autonomic nervous activity, i.e. electrodermal activity (SCRs), and verbal reports were recorded. None of the participants reported motion sickness symptoms. The results showed that (1) SCR was higher for “primed” participants during immersion than initiation in virtual environment (2) “primed” participants had higher SCR than “non primed” in the first virtual tour; “non primed” participants reported higher SCR in the second and third tour than in the first tour; (3) the number of correct intuitive answers was higher in “primed” than “non primed” participants; (4) SCR was higher for “primed” participants “with” than “without” insight when immersed in real environment.

The results provide support for the overarching hypothesis according to which immersion in a real environment with primes would facilitate the rise of intuition and insight in a virtual environment. These results are consistent with the idea that contextual novelty, that is, the naturalistic virtual environment, may be essential for insight^13^. They are also coherent with studies which demonstrate that the identification of consistencies between information provided by an environment influences future decisions^15,22,53–56^. In the current study, the combination of visual and olfactory primes, both firmly associated with “café”, facilitated the identification of a “café” location. However, the results go beyond these findings, as it was demonstrated for the first time, that both intuition and insight can be expressed in a continuum, which, in the current case, starts from a real environment and expands to a virtual environment. Nonverbal reactions (i.e. autonomic reactions) associated with environmental visual and olfactory cues (in a real environment) appear to be the basis from which verbal decisions (i.e. conscious) can be inferred in a virtual environment. Specifically, “primed” participants had higher SCRs when immersed than initiated in the virtual environment. “Primed” participants had higher SCRs than “non primed” participants during the first tour of virtual immersion and gave more accurate intuitive answers than “non primed” participants. These results are consistent with the hypothesis according to which immersion in an environment, whatever its nature, would influence intuitive decisions^5,57,58^. It also supports Bowers et al.^1^ model which states that the assemblage of verbal/nonverbal cues progressively combined in a consistent way would be connected to implicit information. Such processes, qualified as unconscious, would be able to generate representations of the provided information that are accessible to consciousness. In the present study, participants had contrasting experiences since only one group was primed, and as such, only this group would be able to create implicit representations. The autonomic nervous activity of “primed” and “non primed” participants, such as the physiological activity that reflects participants’ unconscious behaviour during real and virtual immersion, was recorded. The observed SCR modifications might mirror participants’ unconscious analysis of the provided sensory information, which, in turn, might facilitate verbal inference of the place to recognise in the virtual environment. Put simply, since SCRs represent an autonomic unconscious activity tractable to cognitive (and emotional) states, the present results would signify that intuitive decisions linked to SCR variations would be understood as a prerequisite indicator of emerging thoughts and feelings consciously expressed when required.

The SCRs of “primed” participants were similar between the three tours but the SCR of “primed” participants was higher than the SCRs of “non primed” participants in the first tour. The SCRs of “non primed” participants were low in the first tour but significantly increased in the second and third tours. However, there is no significant difference between the second and the third tours for “non primed” participants and between “primed” and “non primed” participants for the same tours. This suggests that two different patterns could exist among “primed” and "non primed” participants. For the “primed” ones, the pattern of SCR activation, as sub-cortical and unconscious, is likely to be associated with intuitive decision, while for “non primed” participants, that is not the case, even through the "non primed" participants seem actively try to find the place, as given by the SCRs modifications during the three tours^57–59^. Previous studies have associated modifications in SCRs with conscious or unconscious recognition and a feeling of “knowledge”^29–31^. In the current study, the presence of visual and olfactory primes about “café” in the real environment, and “café” location in the virtual environment could have led to a feeling of “knowledge” for “primed” participants and produced higher SCRs compared to “non primed” participants. This feeling of “knowledge” could be understood as the emerging elaboration of continuity between unconscious and conscious processes, i.e. emerging mental representations. Such a process would pave the way from intuition to insight.

In accordance with Metcalfe and Wiebe^13^, it was considered that the “differentiation of insight problems from other problems by the phenomenology that precedes solution may facilitate illumination of the process of insight”. As such, a phenomenological interview to explore participants’ experience of “insight" was used. During this interview, “primed” and “non primed” participants were asked if they had an understanding of the situation, i.e. an “insight”, when they verbalised their answers. It appeared that more “primed” that “non primed” participants understood the situation. These participants were able to verbalise the expected location (i.e. café) even if they were unconscious of the primes. Such behaviour is consistent with the observed result that primed participants “with” insight showed higher SCR during immersion in the real environment than primed participants “without” insight. Once again, it seems that participants with a higher level of sympathetic activation in the real environment had increased awareness about problem resolution during the virtual experience. It seems that the accumulated “knowledge” of participants, i.e. association of images, essentially based on bi-modal sensory primes, offered participants the possibility to decipher intuition and enact on intuition to produce insight.

In a recent study, Shen et al.^59^ used a verbal insight problem in a real environment while, in the present study a nonverbal insight problem as a link between a real and virtual environment was given. In both studies, insightful participants seem to acquire "knowledge" about a problem without solution, but they unconsciously perceived objects and situations as meaningful. In this study, “primed” participants “with” insight were those who unconsciously understood the link between the information provided in the real and virtual environments. Shen et al.^27^ used “insight” and “non insight” problems and observed greater SCR for the former than for the latter. Similarly, greater SCRs for primed participants “with” insight than for primed participants “without” insight during the immersion in the real environment was observed. Shen et al.^27^ reported an increase of SCR 4 s before problem resolution suggesting that this activation precedes conscious perception of the solution. The current results reinforce this as greater SCR for “primed” participants than “non primed” participants during the first virtual tour was obtained. During this first tour, all participants were invited for the first time to resolve a problem ; specifically to identify a "café" location. “Primed” participants had some cues to the problem solution compared to “non primed” participants. The higher sympathetic activation that was observed during the first tour would suggest the preactivation of a conscious solution or a matching between unconscious primes and conscious perception, which would lead to insight. In this context, “primed” participants had unconsciously built knowledge and representation of the situation, in the real environment, which would be on the basis of the insightful solution in the virtual environment. These results are consistent with the Bowers et al.^1^ model which postulated that intuition and insight can be deployed on the same continuum. They are also consistent with Wallas’ five-stage model where intimations delineated as intuitions are depicted as leading directly to illuminations described as insights^60^. More importantly, and for the first time, these results suggest that this continuum does not only apply to a real environment but to both real and naturalistic virtual environments. Such findings are significantly supported by neurophysiological facts.

Since SCRs are associated with cognitive abstraction, anticipation, and decision making^32,61^ and are connected to subcortical and cortical regions^14^, the implications of SCRs in intuition and insight appear to be predictable. Several studies revealed that superior brain areas involved in intuition and insight such as ventromedial and dorsolateral parts of the prefrontal cortex^18^ are related to SCR^62,63^. In the same vein, additional areas including insula cortex, precuneus and cingulate cortex^64,65^, inferior parietal cortex^20^ and superior temporal cortex^18^ seem to be highly involved in intuition, via an encoding process and insight, and recall strategies^19,28^. In other words, the cognitive approach that underlies the continuum between intuition and insight is facilitated by a specific neurophysiological continuum based on autonomic and central nervous system activities. The implicit knowledge built via the analysis of autonomic areas would be expressed via the intervention of central brain areas.

## Conclusion

The present study put both intuition and insight on a continuum in order to shed light on their relationship. Real and naturalistic virtual environments were used to analyse this continuum. The results support a continuity model according to which bi-modal on the one hand, and unimodal sensory information on the other, i.e. visual and olfactory, and visual respectively, might be an indicator of emerging unconscious representations. Based on these representations (i.e. intuition ingredients) the generation of a correct resolution occurs; that is, insight arises. In the current study, both verbal declarations and autonomic measurements were used via SCR by means of the same paradigm and within the same population to illustrate the continuum between intuition and insight. Moreover, the intuition-insight continuum in a hybrid environment starting from a real situation and extending to a naturalistic virtual one, was analysed. Such an empirical approach might indicate that sympathetic activation through electrodermal activity (i.e. SCRs) would be a physiological indicator of the continuity between past and present representations and would lead to the feeling of “knowledge”, on which insight is based. The continuity between autonomic activity and verbal problem resolution, in terms of the “euréka” moment, would be supported by neurophysiological data as autonomic and central brain activities are neuroanatomically interconnected. Moreover, in this study, the sympathetic activation also contributed to a feeling of familiarity (i.e. being there) between real and virtual environments. Taken together, these findings are coherent with a general view that intuitive judgments in various tasks are associated with the activation of pre-existing implicit knowledge, which is unconsciously retrieved, but nevertheless can elicit an intuitive impression of coherence^1,20,59^ and trigger insight. This might potentially be the source for creative minds and also seems to be consistent with Wallas’ five-stage model in which intuitions appear as intimations that precede illuminations, that is, insights^60,66^. To qualify the dynamic creative process which operates between intuition and insight, Wallas’ concept of "association train" is of interest. Accordingly, intuition is an inkling of an emerging unconscious train of association which may "soar" towards consciousness and engender insight, i.e. euréka. Both intuition and insight are ingenious processes; however, by analysing autonomic activity and verbal expression in a continuum constituted of a hybrid (real and naturalistic artificial) environment, the present study might contribute to a better understanding of the subtleties and nuances of Wallas’ model as well as the interlacing of consciousness and unconsciousness. With the above in mind, intuition and insight are mental representational phenomena involving both cortical and autonomic activities that can occur in continuity in real but also in real/naturalistic virtual environments.
